# The Association between Leisure-Time Physical Activity Intensity and Duration with the Risk of Mortality in Patients with Chronic Kidney Disease with or without Cardiovascular Diseases

**DOI:** 10.31083/j.rcm2307244

**Published:** 2022-06-28

**Authors:** Ning Li, Ruoyang Hong, Weiguo Zhou, Jingchen Zhong, Mingyun Kan, Yawei Zheng, Enchao Zhou, Wei Sun, Lu Zhang

**Affiliations:** ^1^Affiliated Hospital of Nanjing University of Chinese Medicine, Jiangsu Province Hospital of Chinese Medicine, 210029 Nanjing, Jiangsu, China

**Keywords:** chronic kidney disease, leisure-time physical activity, all-cause death, cardiovascular disease

## Abstract

**Introduction:**

For chronic kidney disease (CKD) patients with or without cardiovascular diseases, the associations between leisure-time physical activity intensity (LTPA) and daily exercise time with mortality risk remain unclear.

**Method:**

This study enrolled 3279 CKD patients from National Health and Nutrition Examination Survey (NHANES) 2007–2014 survey. Patients were grouped into different groups according to LTPA intensity (none, moderate, vigorous) and duration (0 min, 0–30 min, 30–60 min, >60 min). We selected the confounders based on their connections with the outcomes of interest or a change in effect estimate of more than 10%. Multivariable-adjusted Cox proportional hazards models were used to examine the associations between LTPA and mortality. The three-knot cubic spline (10, 50, and 90%) was employed to investigate the relationship between the dose of LTPA duration and all-cause death. Patients were divided into different groups according to cardiovascular diseases (CVD).

**Results:**

A total of 564 all-cause death were recorded in this study. Multivariable Cox regression showed that moderate LTPA was associated with a reduced risk of mortality by 38% (hazard ratio (HR): 0.62, 95% confidence interval (CI): 0.44–0.88) in CKD patients, while vigorous LTPA did not have evident survival benefits (HR: 0.91, 95% CI: 0.46–2.64). Subgroups analysis demonstrated that those who engaged in moderate LTPA have a significantly lower risk of mortality (HR: 0.67, 95% CI: 0.47–0.95) in patients without CVD, while patients complicated with CVD did not benefit from the practice (HR: 0.61, 95% CI: 0.37–1.02). Physical exercise for more than 30 minutes was associated with a lower risk of mortality in general CKD patients (30–60 min: HR: 0.23, 95% CI: 0.09–0.58, >60 min: HR: 0.23, 95% CI: 0.08–0.63) and those without CVD (30–60 min/d: HR: 0.32, 95% CI: 0.12–0.83, >60 min/d: HR: 0.20, 95% CI: 0.06–0.71); however, this positive outcome was not seen in patients complicated with CVD (30–60 min/d: HR: 0.67, 95% CI: 0.11–4.04, >60 min/d: HR: 1.14, 95% CI: 0.14–9.11).

**Conclusions:**

Moderate LTPA for more than 30 minutes is associated with a reduced risk of mortality in general CKD patients and those without CVD. However, LTPA did not reduce the risk of mortality in CKD patients complicated with CVD.

## 1. Introduction

Chronic kidney disease (CKD) is a global health concern worldwide. It is currently estimated [1] that nearly 700 million patients worldwide suffer from CKD, which poses a heavy burden on the medical and economic system. CKD patients have a protracted disease course and poor prognosis and are frequently susceptible to numerous organ abnormalities, increasing the risk of death [2]. As one of the top 10 non-communicable causes of death worldwide [3], clinicians are looking for ways to prevent CKD from occurring and developing.

Some novel therapies for CKD treatment have become widely available for clinicians, and new hypoglycemic medications, such as sodium glucose cotransporter-2 inhibitors, have been shown to improve cardiac and renal outcomes in diabetic and non-diabetic CKD patients [4, 5]. In addition to pharmacological therapies, lifestyle interventions such as healthy diets, sodium restriction, and physical activity (PA) are also critical to improving patients’ conditions [6, 7]. For the general population, PA has been proven to reduce the risk of mortality and improve the status of insulin resistance and mental health [8, 9, 10]. Furthermore, some studies demonstrated that PA might reduce the cardiovascular risk of death and improve cardiopulmonary function in CKD patients [11, 12, 13]. Other related research also implied that a certain degree of PA is associated with a reduced risk of mortality and cardiovascular death in CKD patients regardless of diabetes [14]. However, there is currently a lack of studies focusing on the association of leisure-time physical activity (LTPA) intensity and daily duration with the risk of mortality in CKD patients with or without cardiovascular diseases (CVD). There is great heterogeneity among CKD patients [15]. For CKD patients complicated with CVD, their cardiorespiratory fitness is often worse, and the risk of death from CVD and other cerebrovascular diseases is significantly increased [16]. In addition, such patients may be more intolerant of a high-level LTPA that is superior to their own exercise capacity. Therefore, this study aims to conduct a secondary analysis of an observational cohort study based on information collected from the National Health and Nutrition Examination Survey (NHANES) database to investigate the association of LTPA intensity and duration with the risk of all-cause death.

## 2. Method

### 2.1 Study Population

The data of enrolled patients were acquired from the National Health and Nutrition Examination Survey (NHANES) from 2007 to 2014. We chose this period because the investigation questionnaire and laboratory index test method of the variables we are interested in have not changed. The inclusion criteria of our study was estimated glomerular filtration rate (eGFR, eGFR calculation was based on CKD Epidemiology Collaboration equation) <60 mL/min/1.73 $m^{2}$ or urinary albumin-to-creatinine ratio (UACR) >30 mg/g). Those with End stage renal disease (ESRD) (eGFR <15 mL/min/1.73 $m^{2}$ or undergoing dialysis treatment), age <18 years, or without complete follow-up data were excluded from the study. The study procedures of NHANES have been approved by the Institutional Review Board (IRB) of the National Center for Health Statistics (NCHS), with written informed consent obtained. This study obtained permission for ethical exemption from the ethics committee of the Affiliated Hospital of Nanjing University of Traditional Chinese Medicine.

### 2.2 Definition of Physical Intensity and Duration

Data regarding LTPA intensity and duration were obtained from the questionnaires (codebook: PAD660 (How much time do you spend doing vigorous-intensity sports, fitness or recreational activities on a typical day?) and PAD675 (How much time do you spend doing moderate-intensity sports, fitness or recreational activities on a typical day?)). Moderate recreational activities are defined as conducting any LTPA that causes a slight rise in respiration or heart rate, such as brisk walking, bicycling, swimming, or volleyball (moderate exercise corresponds to a metabolic equivalent (MET) score of 4 according to the NHANES suggestion), while vigorous recreational activities are defined as conducting any LTPA that causes a significant increase in respiration or heart rate, such as running or basketball (moderate exercise corresponds to a MET score of 8 according to the NHANES suggestion). Table 1 shows the metabolic equivalent (MET) of various LTPA intensities.

**Table 1.** **Suggested metabolic equivalent (MET) score of relative physical activity intensity**.

Physical activity intensity	Suggested MET score	Example
None	0	Sitting, watching television, lying down
Moderate	4	Brisk walking, cycling, swimming or volleyball
Vigorous	8	Running or basketball

### 2.3 Primary Outcome

All-cause death was the primary prespecified outcome. Mortality and the follow-up time were extracted from the public-use linked mortality file from the National Center for Health Statistics (NCHS) and matched with patients’ IDs from the NHANES database. Given that the NCHS only undated mortality-related data in NHANES from 1999 to 2014, the mortality follow-up time was defined considering those that occurred from the interview date to December 31, 2015 (More details of the mortality file can be found in the following website: https://www.cdc.gov/nchs/data-linkage/mortality-public.htm).

### 2.4 Covariates

Based on results obtained from clinical experience and literature review, the following covariants were selected as the confounding factors in our study: (i) Demographics data: age, sex, race/ethnicity (Mexican American, Non-Hispanic Black, Non-Hispanic White, Other Hispanic, and others); (ii) laboratory index: urinary albumin-to-creatinine ratio (UACR), hemoglobin (Hlb), glycosylated hemoglobin (HbA1c), serum albumin (Alb), aspartate aminotransferase (AST), alanine aminotransferase (ALT), blood urea nitrogen (BUN), serum bicarbonate, serum potassium, serum phosphorous, uric acid (UA), estimated glomerular filtration rate (eGFR (the calculation of eGFR was based on the formula of the Chronic Kidney Disease Epidemiology Collaboration equation (CKD-EPI))); (iii) chronic diseases: CVD (complications such as heart failure, coronary heart diseases, or with a history of heart attack), hypertension, diabetes, smoking status (never, used to smoke, current smoker), and body mass index (BMI).

### 2.5 Statistical Analysis

The statistical analysis and weight of the study population calculations (calculated as 1/4 × 2-year follow-up weight of total samples) followed the NHANES recommended analytical guidelines [17]. For the description of participants, we group the enrolled patients into three groups (none/moderate/vigorous) according to the LTPA intensity. Shapiro-Wilk method was utilized to examine the normal distribution of continuous data. The constant variables conformed to normal distribution were compared using an Analysis of Variance (ANOVA) test and are presented as mean ± standard deviation (SD).

Subsequently, Kruskal-Wallis (KW) test was used to determine the non-normally distributed variables, and they were presented as median (1st–3rd quartile). Chi-square tests were performed to compare categorical variables. Fisher’s exact test was used if the theoretical frequency was less than 10. We defined LTPA intensity as the key independent variable and all-cause death as the dependent variable. Univariate, multivariate-adjusted Cox proportional hazards model and Kaplan-Meier (KM) curve were used to explore the associations of the LTPA intensities with mortality risks due to all causes. Furthermore, we adjusted for the covariates that changed the matched hazard ratio by at least 10 percent when added to the model. For the duration of LTPA, we divided them into four groups (0 min, 0–30 min, 30–60 min, and ≥60 min). KM curve, univariate, and multivariable Cox regression outlined the association between LTPA duration and mortality risk. The three-knot cubic spline [18] (10, 50, and 90%) was employed to investigate the relationship between the dose of LTPA duration and all-cause death. All analyses were repeated among various subgroups (presence or absence of CVD). The statistical analyses were performed using R software version 4.05 (https://www.rproject.org/), and *p *< 0.05 (double) was considered statistically significant.

## 3. Results

### 3.1 Baseline Characteristics

This study enrolled a total of 3279 CKD patients. Among them, 2139 patients did not participate in any form of LTPA; 832 patients engaged in moderate LTPA; and 308 involved in vigorous LTPA. Table 2 shows that higher levels of LTPA intensity were prone to have lower ages, proteinuria, glycosylated hemoglobin, BUN, UA, BMI, heart failure, coronary heart disease, diabetes, anemia, smoking rate, and mortality (*p *< 0.01). In addition, those with greater levels of LTPA intensity were considered prone to have higher hemoglobin and eGFR levels (*p *< 0.01).

**Table 2.** **Baseline characteristics of enrolled patients**.

Variables		None (n = 2139)	Moderate (n = 832)	Vigorous (n = 308)	*p*-value
Age (years)		63.52 (62.67, 64.38)	59.44 (57.87, 61.00)	46.07 (43.47, 48.66)	<0.0001
UACR (mg/g)		169.44 (142.57, 196.32)	152.97 (112.54, 193.40)	101.23 (75.62, 126.83)	0.0011
Hemoglobin (g/dL)		13.74 (13.64, 13.83)	13.84 (13.69, 13.98)	14.09 (13.92, 14.26)	0.0015
Glycosylated hemoglobin (g/dL)		6.26 (6.18, 6.34)	6.01 (5.91, 6.11)	5.76 (5.61, 5.91)	<0.0001
Serum albumin (g/dL)		41.26 (41.07, 41.44)	42.14 (41.86, 42.42)	43.37 (42.94, 43.80)	<0.0001
AST (U/L)		27.09 (25.69, 28.48)	25.74 (24.74, 26.75)	28.94 (24.12, 33.76)	0.1698
ALT (U/L)		24.53 (22.68, 26.37)	23.31 (22.18, 24.43)	25.69 (22.85, 28.53)	0.221
BUN (mmol/L)		6.51 (6.34, 6.69)	6.03 (5.80, 6.26)	5.17 (4.88, 5.46)	<0.0001
Serum bicarbonate (mmol/L)		25.03 (24.89, 25.16)	25.09 (24.86, 25.31)	25.29 (24.99, 25.60)	0.2969
Serum potassium (mmol/L)		4.08 (4.06, 4.11)	4.06 (4.02, 4.09)	4.01 (3.96, 4.06)	0.0224
Serum phosphrous (mmol/L)		1.23 (1.22, 1.24)	1.23 (1.21, 1.25)	1.23 (1.20, 1.26)	0.9642
UA (umol/L)		366.17 (360.89, 371.44)	351.90 (343.65, 360.15)	339.25 (325.75, 352.75)	0.0001
BMI (Kg/$m^{2}$)		30.79 (30.35, 31.23)	29.04 (28.44, 29.64)	27.24 (26.38, 28.11)	<0.0001
eGFR (mL/min/1.73 $m^{2}$ )		68.99 (67.46, 70.52)	72.96 (70.57, 75.35)	85.62 (81.72, 89.52)	<0.0001
Gender (%)					0.0622
	Male	59.59	58.33	50.84	
	Female	40.41	41.67	49.16	
Race/Ethnicity (%)					0.0225
	Mexican American	7.65	4.9	8.4	
	Non-Hispanic Black	12.84	9.87	12.04	
	Non-Hispanic White	69.07	74.83	67.96	
	Other Hispanic	5.04	4.28	4.89	
	Other Race	5.40	6.12	6.71	
Heart failure (%)					<0.0001
	No	89.29	93.12	99.01	
	Yes	10.71	6.88	0.99	
Coronary heart diseases (%)					0.0029
	No	89.84	90.16	96.8	
	Yes	10.16	9.84	3.2	
Hypertension (%)					<0.0001
	No	27.51	38.96	64.92	
	Yes	72.49	61.04	35.08	
Diabetes (%)					<0.0001
	No	63.89	76.16	85.05	
	Yes	36.11	23.84	14.95	
Mortality (%)					<0.0001
	No	81.56	92.43	96.5	
	Yes	18.44	7.57	3.5	
Anemia (%)					0.0005
	No	83.04	85.86	92.33	
	Yes	16.96	14.14	7.67	
Smoking status (%)					<0.0001
	Never	46.38	51.86	65.96	
	Former	34.04	33.93	23.99	
	Currently	19.57	14.21	10.05	

Values for categorical variables are given as percentages. The expression form of continuous variables is mainly presented as median (1st–3rd quartile) due to their non-normally distributed. Abbreviations: UACR, albumin-to-creatinine ratio; AST, aspartate aminotransferase; ALT, alanine aminotransferase; BUN, blood urea nitrogen; UA, uric acid; BMI, body mass index; eGFR, estimated glomerular filtration rate.

### 3.2 LTPA Intensity and All-Cause Death

**Fig. 1.**
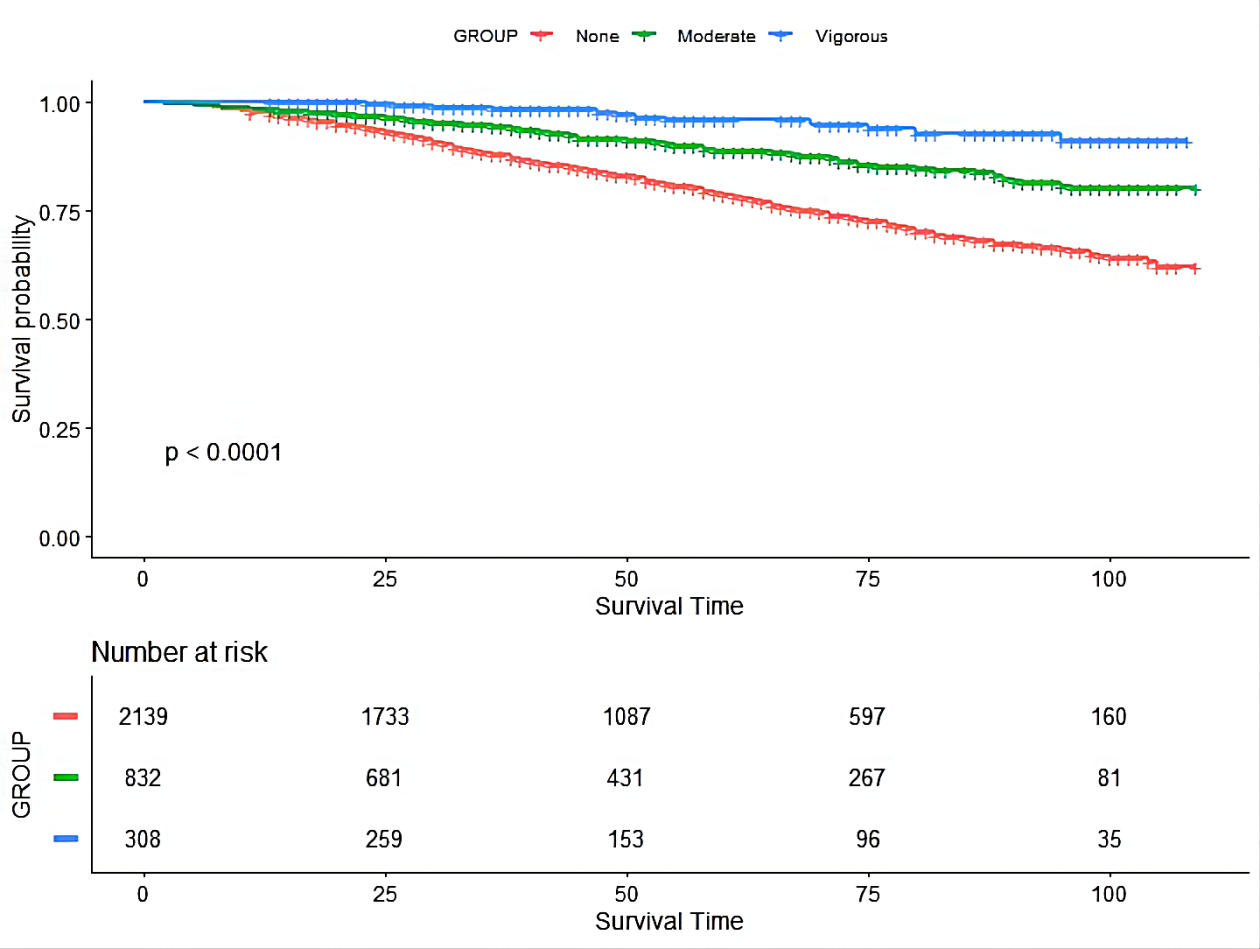
**The Kaplan-Meier (KM) curve of the association between leisure-time physical activity intensity with mortality risk in chronic kidney disease patients**.

During a median 120-month follow-up, 564 all-cause death were recorded. The KM curve (Fig. 1) indicated that moderate and vigorous LTPA intensity is associated with lower risks of mortality compared with those who never exercise (*p *< 0.01). Furthermore, univariable Cox regression (Table 3) showed that moderate LTPA is associated with a reduced risk of all-cause death by 62% (hazard ratio (HR): 0.38, 95% confidence interval (CI): 0.29–0.50, *p *< 0.01). In patients exposed to vigorous LTPA, the rate was reduced even more by 82% (HR: 0.18, 95% CI: 0.08–0.39, *p *< 0.01). After adjusting age, Alb, BUN, UA, BMI, hypertension, eGFR, and LTPA duration (according to a change in effect estimate of more than 10%), moderate LTPA remained associated with a lower risk of all-cause death (HR: 0.62, 95% CI: 0.44–0.88, *p *< 0.01), while vigorous LTPA did not have a significant effect on it (HR: 1.10, 95% CI: 0.46–2.64, *p* = 0.83).

**Table 3.** **Association between leisure-time physical activity intensity (LTPA) and risk of mortality in CKD patients**.

	Leisure-time physical activity intensity		
	None	Moderate	Vigorous
Participants, N	2139	832	308
Model 1 (HR, 95% CI)	1.00 (Reference)	0.38 (0.29–0.50)	0.18 (0.08–0.39)
Model 2 (HR, 95% CI)	1.00 (Reference)	0.62 (0.44–0.88)	1.10 (0.46–2.64)

Values for categorical variables are given as percentages. The expression form of continuous variables is mainly presented as median (1st–3rd quartile) due to their non-normally distributed. Model 1: unadjusted model; Model 2: adjusted for age, serum albumin, blood urea nitrogen, uric acid, body mass index, hypertension, estimated glomerular filtration rate, and LTPA duration (according to a change in effect estimate of more than 10%).

### 3.3 Subgroup Analysis for LTPA Intensity

**Fig. 2.**
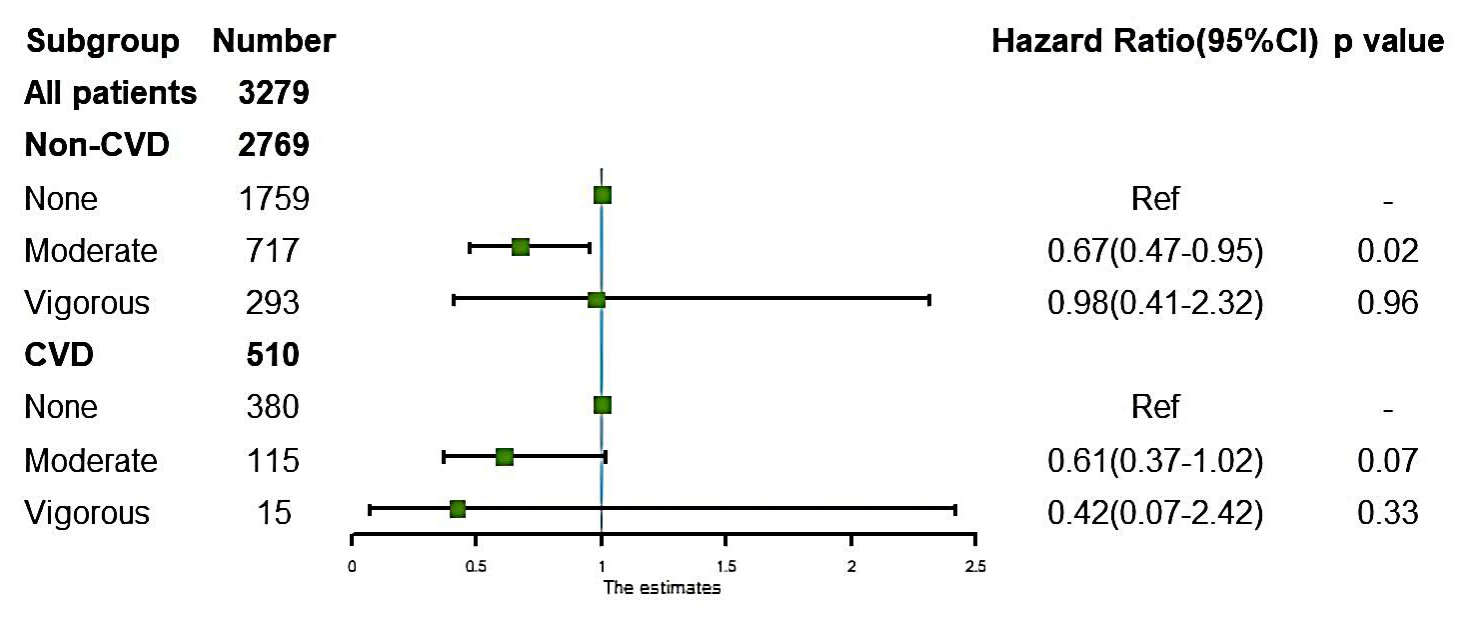
**The association of leisure-time physical activity intensity and mortality risk in chronic kidney disease patients with or without cardiovascular diseases (CVD)**. Adjusted for age, serum albumin, blood urea nitrogen, uric acid, body mass index, hypertension, estimated glomerular filtration rate, and LTPA duration. CVD: complications such as heart failure, coronary heart disease, or a history of heart attack.

The subgroup analysis (Fig. 2) showed that moderate LTPA reduced the risk of all-cause death by 33% in patients without CVD (HR: 0.67, 95% CI: 0.47–0.95, *p* = 0.02). However, for patients with complicated conditions with CVD, moderate LTPA did not have significant benefits (HR: 0.61, 95% CI: 0.37–1.02, *p* = 0.07). Vigorous LTPA did not improve the survival outcomes regardless of the presence of CVD (non-CVD: HR: 0.98, 95% CI: 0.41–2.32, *p* = 0.96; CVD: HR: 0.42, 95% CI: 0.07–2.42, *p* = 0.33).

### 3.4 LTPA Duration and All-Cause Death

**Fig. 3.**
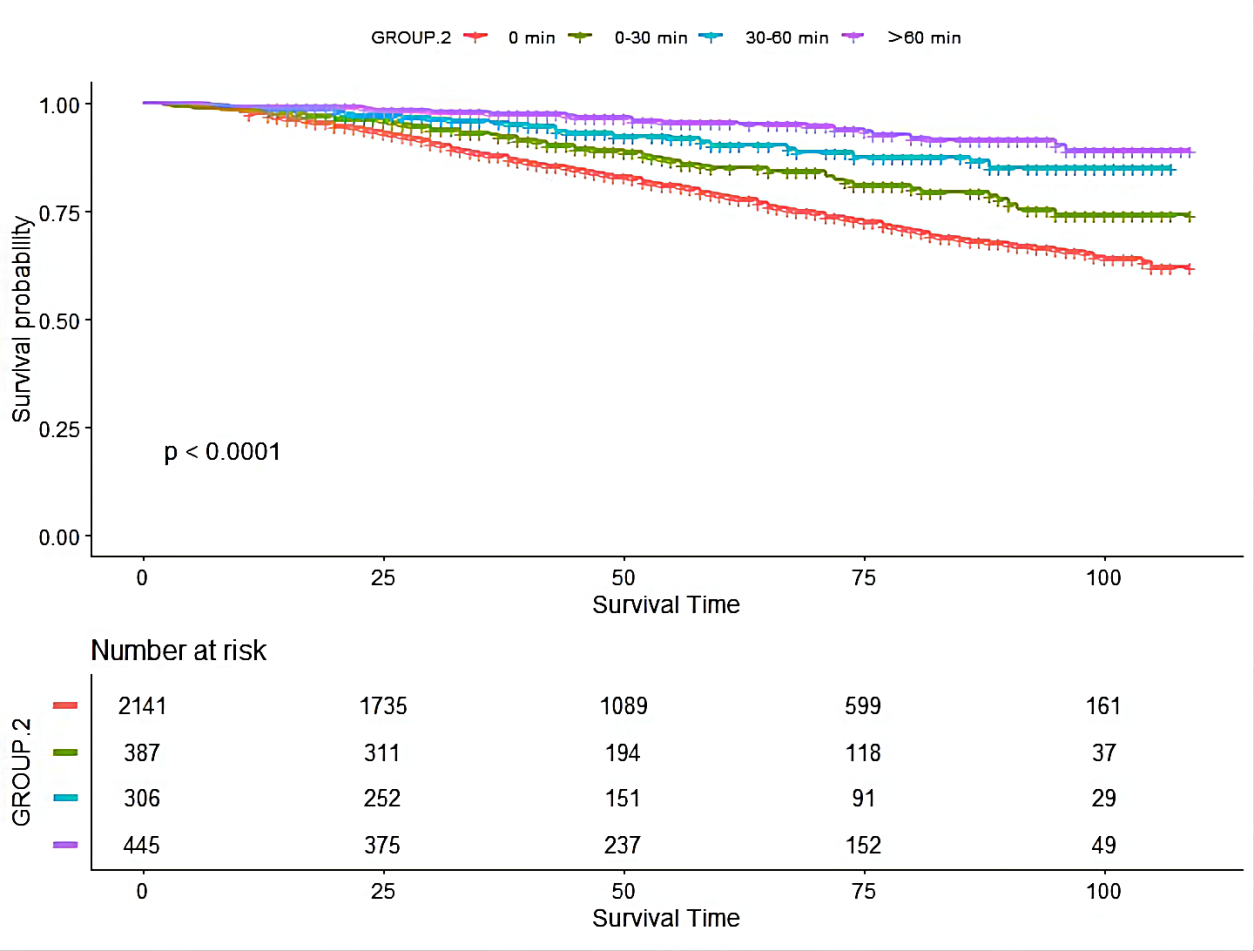
**The Kaplan-Meier (KM) curve of the association between leisure-time physical activity duration with mortality risk in chronic kidney disease patients**.

**Fig. 4.**
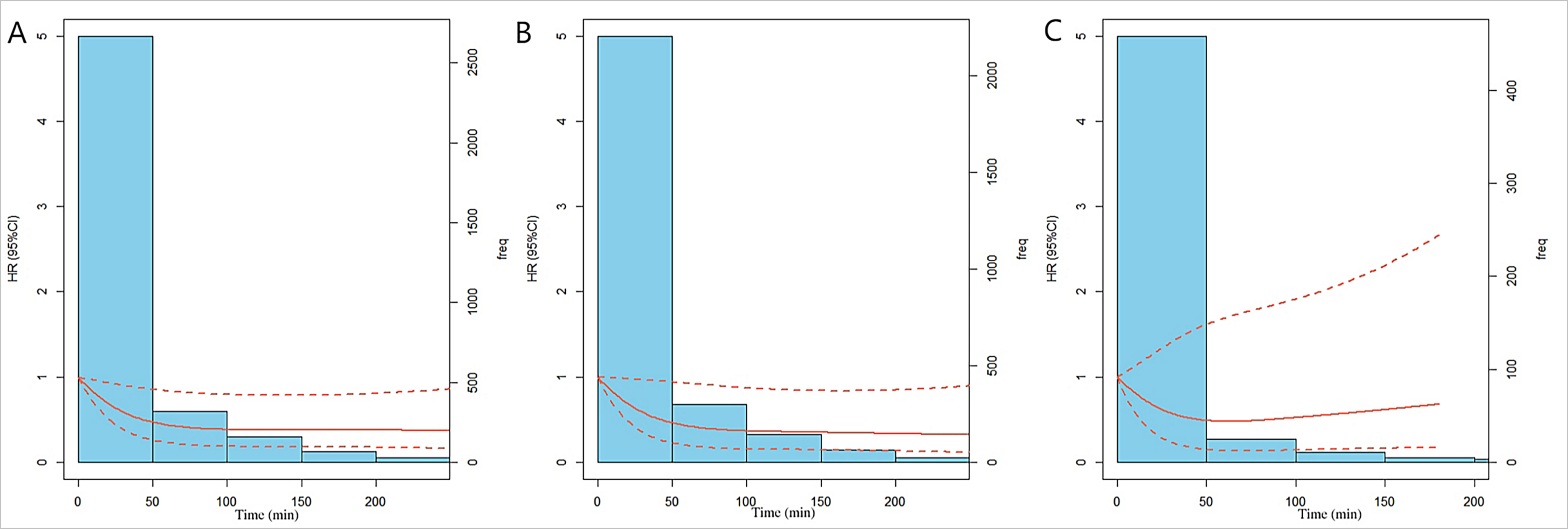
**Relationship between the dose of leisure-time physical activity duration and the risk of mortality in CKD patients, according to the three-knot cubic spline (10, 50, and 90%)**. The solid line represents the multivariable adjusted hazard ratios (adjusted for sex, blood urea nitrogen, serum potassium, heart failure, hypertension, anemia, and estimated glomerular filtration rate.), with dashed lines showing that 95% of confidence intervals derive from restricted cubic spline regression with three knots. The ordinate value of the dashed lines across 1.0 indicates no significant difference compared with the reference point (0 min). (A) general CKD patients. (B) patients without cardiovascular disease. (C) patients complicated with cardiovascular disease (complications such as heart failure, coronary heart diseases, or with a history of heart attack).

KM curve (Fig. 3) indicated that the groups who engaged in different physical exercises consistently showed a lower risk of death than those who didn’t. Similar effects could be seen when using univariable Cox regression (Table 4) (0–30 min/d: HR: 0.55, 95% CI: 0.39–0.78, *p *< 0.01; 30–60 min/d: HR: 0.27, 95% CI: 0.17–0.42, *p *< 0.01; >60 min/d: HR: 0.20, 95% CI: 0.11–0.36, *p *< 0.01). After adjusting sex, BUN, potassium, heart failure, hypertension, anemia, and eGFR (according to a change in effect estimate of more than 10%), those who had an LTPA duration of more than 30 minutes had a significant survival advantage (30–60 min/d: HR: 0.23, 95% CI: 0.09–0.58, *p *< 0.01; >60 min/d: HR: 0.23, 95% CI: 0.08–0.63, *p *< 0.01). Nonetheless, LTPA lasting less than 30 min/d did not reduce the risk of all-cause death (HR: 0.44, 95% CI: 0.19–1.01, *p* = 0.06). Restricted cubic splines showed that when the LTPA duration reached 110 min/d, the risk of death tended to cause a plateau effect (Fig. 4).

**Table 4.** **The association between leisure-time physical activity (LTPA) duration and the risk of mortality in CKD patients**.

	Leisure-time physical activity duration			
	0 min/d	0–30 min/d	30–60 min/d	>60 min/d
Participants, N	2181	387	306	445
Model 3	1.00 (Reference)	0.55 (0.39–0.78)	0.27 (0.17–0.42)	0.20 (0.11–0.36)
Model 4	1.00 (Reference)	0.44 (0.19–1.01)	0.23 (0.09–0.58)	0.23 (0.08–0.63)

Model 3: unadjusted model; Model 4: adjusted for sex, blood urea nitrogen, serum potassium, heart failure, hypertension, anemia, and estimated glomerular filtration rate (according to a change in effect estimate of more than 10%).

### 3.5 Subgroup Analysis for LTPA Duration

**Fig. 5.**
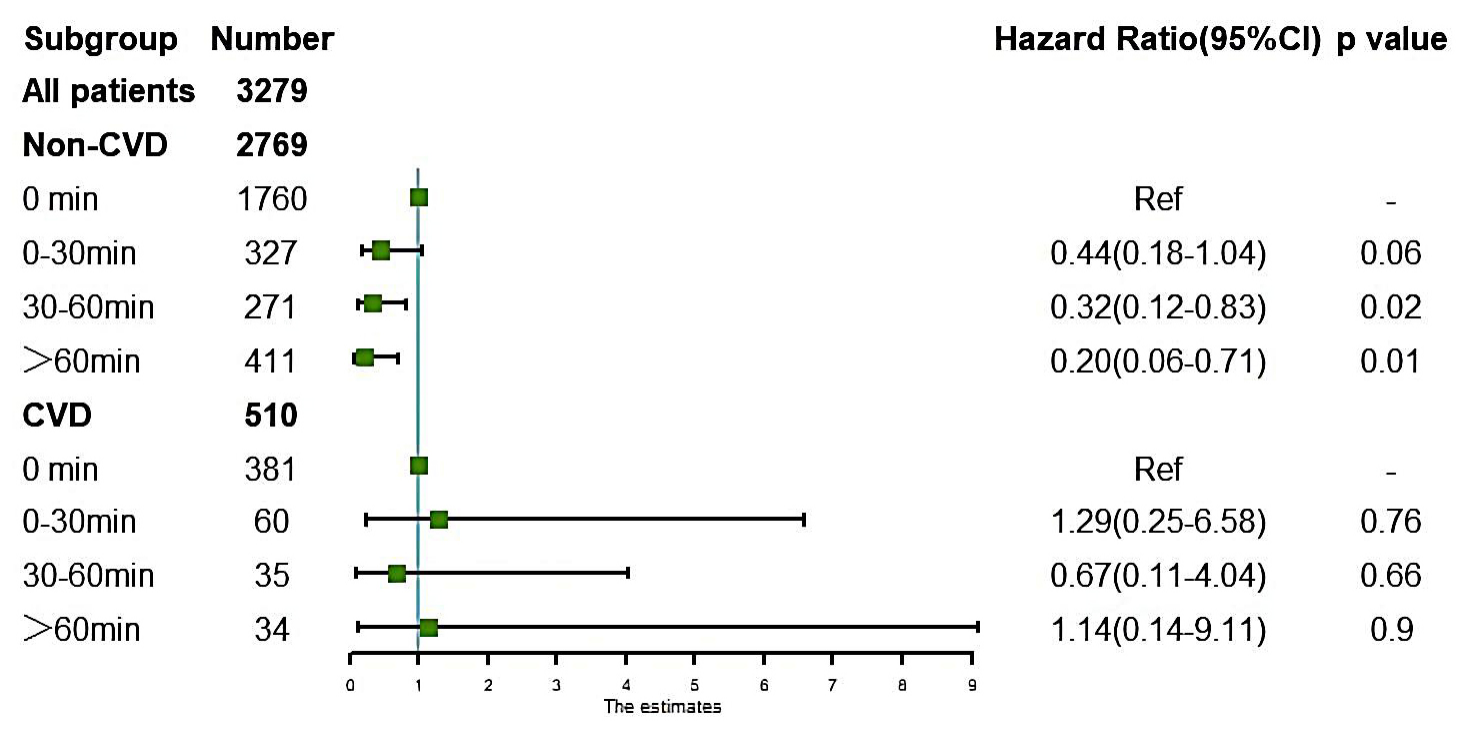
**Association of leisure-time physical activity duration and the risk of mortality in chronic kidney diseases patients with or without cardiovascular disease (CVD)**. Adjusted for sex, blood urea nitrogen, serum potassium, heart failure, hypertension, anemia, and estimated glomerular filtration rate. CVD: complications such as heart failure, coronary heart disease, or a history of heart attack.

Patients who practiced LTPA for more than 30 minutes per day had a lower risk of all-cause death in patients without CVD (Fig. 5) (0–30 min/d: HR: 0.44, 95% CI: 0.18–1.04, *p* = 0.06; 30–60 min/d: HR: 0.32, 95% CI: 0.12–0.83, *p* = 0.02; >60 min/d: HR: 0.20, 95% CI: 0.06–0.71, *p* = 0.01). Restricted cubic splines demonstrated that the risk of death decreased with the extension of exercise time and reached the plateau effect when the daily rate was more than 110 min/d (Fig. 4). For patients with CVD complications, there was no significant survival benefit at any time period (0–30 min/d: HR: 1.29, 95% CI: 0.25–6.58, *p* = 0.76; 30–60 min/d: HR: 0.67, 95% CI: 0.11–4.04, *p* = 0.66; >60 min/d: HR: 1.14, 95% CI: 0.14–9.11, *p* = 0.9). Although the relative risk decreased with longer times of LTPA, each period did not reach statistical significance, according to the restricted cubic spline (Fig. 4).

## 4. Discussion

PA has been demonstrated to improve cardiorespiratory fitness and reduce the risk of cardiovascular and renal outcomes in the general population [9, 19]. According to the guidelines from the Kidney Disease: Improving Global Outcomes (KDIGO), physical activities should be recommended as a routine treatment for patients with CKD [20]. Nevertheless, the association of LTPA intensity and daily exercise time with mortality risk in CKD patients complicated with CVD remains unclear.

Our study is the first to focus on investigating the survival benefits of LTPA in patients with CVD. We found that moderate LTPA is associated with a reduced risk of mortality for general CKD patients. However, vigorous LTPA did not have any significant benefits in their conditions. These results raise questions about whether patients with CKD can benefit from vigorous exercise. We assume that there might be different reasons behind our primary results. First, the inclusion of some covariates (e.g., eGFR and age, which may reflect the progression of diseases and is associated with a certain level of mortality) may modify the benefit of LTPA on CKD patients. Secondly, the number of patients who exercised vigorously was considerably smaller than the samples in other studies, which may cause a particular risk of bias. It is important to mention that several studies also have similar results as us, Beddhu *et al*. [12] conducted a study based on the NHANES database and found that light-intensity LTPA may help reduce the risk of mortality in CKD patients. while moderate or vigorous intensity was not associated with a lower risk of death. Differently, Kim J. *et al*. [21] demonstrated that a low and moderate amount of PA was associated with a lower risk of all-cause death; however, vigorous exercise still did not have considerable benefits. It is unclear whether vigorous PA intensity can help improve the prognosis of CKD patients. Given most of the research was observational and relied on self-reported data, further high-quality studies are needed to validate the association between vigorous PA intensity and potential benefits for patients diagnosed with CKD.

Cardiovascular diseases are the leading cause of death in patients with CKD [16, 22], which sheds light on the importance of a reasonable PA prescription for patients with CVD complications. Our subgroup analysis identified different survival benefits for patients with or without CVD. LTPA did not see a clear survival benefit for patients complicated with CVD, suggesting that physical exercise may have individual differences in the survival benefits of the CKD population. It’s worth mentioning that research on the impact of LTPA on these patients has been limited. Some studies have demonstrated that regular exercise can reduce the risk of cardiovascular and all-cause death in individuals with heart failure [23, 24]. Furthermore, several studies also revealed that higher levels of PA were associated with a lower risk of mortality in patients with coronary artery calcification [25]. Nonetheless, these studies did not analyze the subgroups of patients with CKD. According to our research, LTPA may not bring survival benefits for CKD patients complicated with CVD. However, our results also pointed out that moderate LTPA reduced mortality risk in patients without cardiovascular disease, showing that physical exercise may have varying survival benefits for CKD patients with various baseline characteristics. In the future, more high-quality studies are needed to find out how physical activity affects patients with CKD with different individual features.

The ideal duration of daily physical activity for those with CKD is still unclear. Our study found that LTPA for more than 30 minutes per day is associated with a reduced risk of mortality for general CKD patients. However, when the time went beyond 110 min/d, the relative risk value reached a plateau effect. Previously, an observational study conducted by Zhang NH pointed out that the risk of mortality was relatively increased in CKD patients who reported more than 900 min/week of LTPA. However, it was not statistically significant [11]. In light of these findings, we propose that the dose of LTPA should be reasonably controlled for patients with CKD rather than being excessive or too low.

It is noteworthy that different exercise periods did not diminish the mortality risk in CKD patients with CVD. This demonstrates that LTPA does not improve survival outcomes in CKD patients complicated with CVD, which is consistent with the findings related to LTPA intensity in our study. We assume that this may be due to the high heterogeneity among CKD patients [15] and the critical condition of patients complicated with CVD, since most patients with CVD are exposed to a higher risk of disease progression and all-cause mortality [16]. In contrast, patients without CVD had better tolerance to physical exercise. Our results showed that, in these cases, LTPA for more than 30 minutes per day was associated with reduced mortality risk. Our outcomes challenge the results presented by Kim MH in a previous study that indicated that longer weekly periods of PA were associated with lower risks of all-cause death in the general population, regardless of whether or not they had CVD [26]. The varying conclusions may be because the enrolled patients in our study were different since the proportion of CKD in that study only accounted for 5%, and those who had their conditions complicated with CVD had a lower mean eGFR level (53 versus 67 mL/min/1.73 $m^{2}$). In addition, the survey method we applied focuses on the patient’s weekly fixed exercise intensity and frequency rather than the exercise level recalled in the past 7 days, so the results are more reliable. According to our observations, patients with CKD who engage in at least 30 minutes of moderate LTPA each day are associated with lower mortality risk. This dose is also in line with the standard recommendation of the KDIGO guideline.

It is equally important to acknowledge the limitations of our study. First, the population enrolled in our study involved mainly stage 1–4 CKD individuals, while stage 5 CKD patients and those who undergo dialysis (who are high-risk groups for CVD complications) still need to be included in more detailed research. Second, since CKD patients are exposed to weakened cardiopulmonary systems and prone to a high level of frailty index, they may have a lower PA capacity compared with healthy individuals. The use of MET as a metric for assessing exercise intensity may result in some bias. This is also the limitation of most current observational studies. Future studies should focus on using an individual’s PA capacity as a way to measure the intensity of PA. Third, most of the baseline characteristics of the various LTPA intensity groups were different. Although we were dedicated to controlling the confounding factors in the regression model, there may still be other factors interfering with the reliability of the results.

## 5. Conclusions

We have concluded that moderate LTPA for more than 30 minutes is directly associated with a reduced risk of mortality in general CKD patients. This advantage was also evident in individuals without CVD. Nonetheless, LTPA was not associated with an improved survival outcome in CKD patients complicated with CVD. Our findings indicate that the survival benefit of LTPA in patients with CKD appears to vary depending on the individual with or without CVD, emphasizing the significance of tailoring PA prescriptions to the individual characteristics of the CKD population.

## Data Availability

The datasets used and analyzed in this study are available from the first author and corresponding author on reasonable request.
